# Switching from immediate- to extended-release cysteamine in patients with nephropathic cystinosis: from clinical trials to clinical practice

**DOI:** 10.1093/ckj/sfae049

**Published:** 2024-03-06

**Authors:** Gema Ariceta, Fernando Santos, Andrés López Muñiz, Alvaro Hermida, Maria Luisa Matoses, Ana Ventura, Paloma Leticia Martin-Moreno, Esther González, Laura Acuña, Elisa Giner, Julia Vara

**Affiliations:** Paediatric Nephrology Department, Hospital Vall d’Hebrón, Autonomous University of Barcelona, Barcelona, Spain; Paediatric Nephrology Department, Hospital Universitario Central de Asturias, University of Oviedo, Oviedo, Spain; Nephrology Department, Complejo Hospitalario Universitario de la Coruña, A Coruña, Spain; Department of Internal Medicine, University of Santiago de Compostela, Santiago de Compostela, Spain; Paediatric Nephrology Department, Hospital Universitario La Fe, Valencia, Spain; Nephrology Department, Hospital Universitario La Fe, Valencia, Spain; Nephrology Department, Clinica Universidad de Navarra, Navarra Institute for Health Research, Pamplona, Spain; Nephrology Department, Hospital 12 de Octubre, Madrid, Spain; Medical Department, Chiesi España S.A.U., Barcelona, Spain; Medical Department, Chiesi España S.A.U., Barcelona, Spain; Paediatric Nephrology Department, Hospital 12 de Octubre, Madrid, Spain

**Keywords:** effectiveness, extended-release cysteamine, immediate-release cysteamine, nephropathic cystinosis, switching

## Abstract

**Background:**

The purpose of this study is to evaluate the effectiveness and safety of switching from immediate-release (IR) to extended-release (ER) cysteamine in patients with nephropathic cystinosis (NC) in Spain.

**Methods:**

We conducted an observational, retrospective, multicentre study in NC patients who received IR cysteamine for at least 12 months, switched to ER cysteamine, and received it for at least 6 months before inclusion.

**Results:**

Data were collected from nine patients (four children, five adults) 36 months before and after the switch. Despite the highly selected population, an improvement in growth, particularly in children and a significant reduction in hospitalization days was observed. A decrease in halitosis, body odour and gastrointestinal effects was reported in most of the patients who suffered before the switch, and the use of proton pump inhibitors (PPIs) decreased in some patients. The estimated glomerular filtration rate (eGFR) remained stable in patients with preserved kidney function. No significant changes in white blood cell (WBC) cystine levels were observed after the switch. There was no significant difference in the cysteamine dose received. However, some patients were receiving <50% of the recommended dose of cysteamine before and after the switch and showed elevated levels of WBC cystine.

**Conclusions:**

Switching from IR to ER cysteamine in clinical practice reduces hospital stays, improves nutritional status and growth in paediatric patients and could help to enhance treatment tolerability by reducing side effects. Furthermore, the dosing of ER cysteamine could promote therapeutic compliance and positively affect the quality of life of the NC population.

## Graphical Abstract

**Graphical Abstract:**
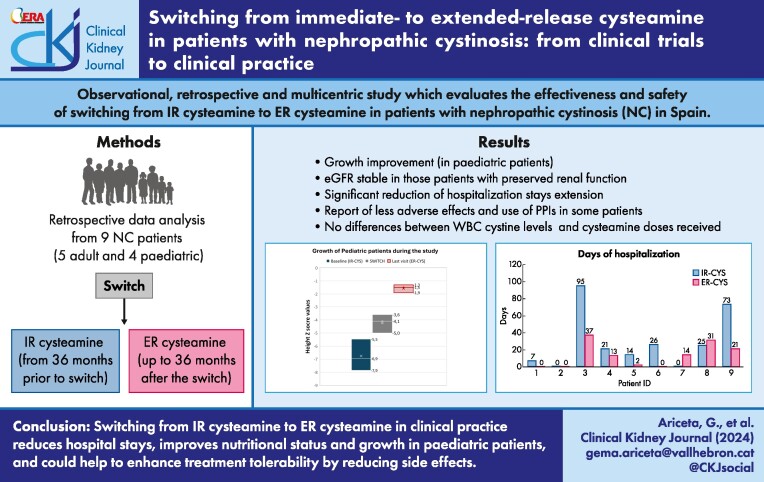

KEY LEARNING POINTS **What was known:**

- Nephropathic cystinosis (NC) is an ultrarare lysosomal disease that leads to the progressive deterioration of multiple organs, especially the kidney. Cystine depletion therapy or cysteamine is the mainstream lifelong treatment for NC. Correct therapeutic compliance is key to preventing and delaying progression of the disease.
- Two formulations of cysteamine are available: immediate release (IR) and extended release (ER). IR cysteamine must be taken on a strict schedule every 6 hours, which prevents a continuous night's rest. ER cysteamine is administered every 12 hours, providing a better treatment schedule and uninterrupted sleep.
- Although several studies have shown the effectiveness of ER cysteamine in controlling disease progression and improving the quality of life of patients, more results under routine clinical practice are needed to increase the evidence of the benefit of switching from IR to ER cysteamine.

**This study adds:**

- The growth of paediatric patients has been improved significantly after switching from IR to ER cysteamine. The duration (in days) of hospitalization decreased significantly after switching to ER cysteamine. There was a reduction in the reports of adverse effects and in the use of proton pump inhibitors when patients switched to ER cysteamine.
- ER cysteamine treatment could be beneficial for all patients with NC, delaying disease progression, improving their clinical status and providing better tolerability.

**Potential impact:**

- Switching from IR to ER cysteamine in clinical practice could help to improve tolerability and reduce hospital stays in patients with NC, as well as achieve optimal nutritional status and growth in paediatric patients. Furthermore, the dosing of ER cysteamine could promote therapeutic compliance and positively impact the patient's quality of life.

## INTRODUCTION

Nephropathic cystinosis (NC) is an ultrarare autosomal recessive metabolic disorder with an estimated incidence of 1:100 000–200 000 live births. Different mutations in the *CTNS* gene encoding the lysosomal transport protein cystinosin result in a non-functional transporter and cystine accumulation within the lysosomes [1], leading to the progressive deterioration of multiple organs, especially the kidney [2]. Diagnosis is confirmed by quantification of increased white blood cell (WBC) cystine levels (≥1 nmol hemicystine/mg protein in untreated patients) and/or confirmatory genetic testing.

Cystine depletion therapy (CDT) with cysteamine represents the mainstay of treatment in NC. It has been extensively demonstrated that CDT should start immediately after diagnosis and continue throughout life, which leads to improved growth, preservation of renal and extrarenal organ function and increased life expectancy [3–6]. In the absence of CDT, kidney failure occurs in the first decade of life [4, 7].

Unfortunately, cysteamine causes significant side effects, including gastrointestinal (GI) symptoms, halitosis and unpleasant body odour. All of these negatively impact patient compliance and may compromise clinical outcomes [8–10].

Two oral formulations of cysteamine are currently available: immediate-release (IR) cysteamine and extended-release (ER) cysteamine. Both formulations have been shown to be effective in reducing WBC cystine levels and preserving kidney function, but IR cysteamine must be taken on a strict schedule every 6 hours, which prevents a continuous night rest and significantly affects the quality of life (QoL) of patients and caregivers.

ER cysteamine is administered every 12 hours, providing a better treatment schedule and uninterrupted sleep. ER cysteamine has demonstrated non-inferiority compared with IR cysteamine, allowing an 18% reduction in the total daily dose of cysteamine in patients with NC [11]. Further, 24 months of ER cysteamine treatment has been shown to maintain WBC cystine levels, provide stable growth and kidney function and benefit patient's QoL compared with treatment with IR cysteamine [12]. Recent results also demonstrate the efficacy and safety of ER cysteamine in treatment-naïve children <6 years of age who, after 18 months of receiving ER cysteamine, exhibited a significant decrease in WBC cystine levels and an improvement in growth and glomerular filtration rate (GFR) [13].

Regarding safety, an additional study showed that ER cysteamine treatment was not only as effective as IR cysteamine treatment, but was also associated with fewer adverse effects [14], which could imply better tolerability to ER cysteamine, and thus a potential tool to improve treatment adherence [15]. Good therapeutic compliance is a key factor in the management of NC and is essential to prevent end-organ damage and improve the prognosis of affected patients [16]. Adherence to IR cysteamine treatment is good in paediatric patients (94%) but decreases over time to ≈50% in adolescents and adults [17]. In this regard, a recent study using an electronic medication event monitoring system in patients with NC demonstrated better overall timely coverage by the action of ER cysteamine versus IR cysteamine (22.8 h versus 14.9 h on a daily basis) [15].

Despite the benefits of ER cysteamine mentioned above, there are currently major access problems in Spain, as this drug is not included in the basic portfolio of the National Health System (NHS). Furthermore, there are very few published series related to NC and its management in real clinical practice. This study aims to analyse the experience of switching from IR cysteamine to ER cysteamine in patients with NC under routine clinical practice conditions in Spain.

## MATERIALS AND METHODS

### Study design

This observational, retrospective, multicentre study was conducted in seven hospitals in Spain by nine investigators. The study protocol fulfilled the ethical principles of the Declaration of Helsinki, the standards of Good Clinical Practice and applicable legislation and was approved by the Ethics Committee of the Hospital 12 de Octubre, Madrid, Spain (CHI-CIS-2020-01). Written informed consent was obtained from all participants before data collection.

**Figure 1:**
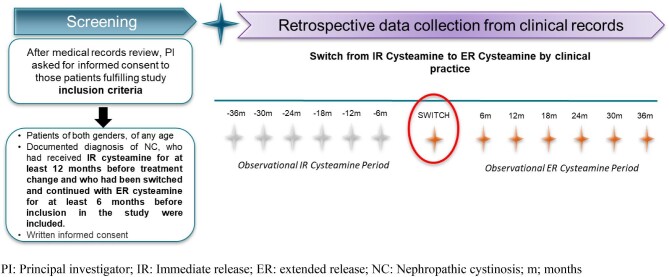
Scheme of the study design.

The study consisted of a single visit where the patient's medical records data were collected for all visits from 36 months before to 36 months after switching from IR to ER cysteamine (including the day of the switch) and following standard clinical practice (Fig. 1).

### Study population

Patients of any age, with a documented diagnosis of NC, who had received IR cysteamine for at least 12 months before the switch to ER cysteamine and for at least 6 months before inclusion in the study participated after signing consent/assent (Fig. 1). The decision to switch the treatment was completely dissociated from enrolment in this study and was made by the treating physician based on the patient’s clinical evaluation.

### Study objectives

The objective of this study was to determine the benefits of switching from IR cysteamine (Cystagon, Recordati Rare Diseases, Milam, Italy) to ER cysteamine (Procysbi, Chiesi Farmaceutici SpA, Parma, Italy) treatment under standard routine clinical practice. The primary efficacy endpoints assessed were WBC cystine levels, cysteamine total daily dose, deterioration of kidney function based on estimated glomerular filtration rate (eGFR) trend (using the modified Schwartz formula [18] in paediatric patients and the Chronic Kidney Disease Epidemiology Collaboration (CKD-EPI) formula [19] in adult patients), growth based on the Z score and the reason for switching. Furthermore, the duration and the number of hospitalizations (in terms of frequency) were evaluated as secondary efficacy endpoints and descriptive variables related to the disease and treatment were registered, including kidney transplantation (KT) and extrarenal manifestations. Regarding safety and tolerability assessment, the comparison of the incidence of GI side effects, use of proton pump inhibitors (PPIs) and the presence of body odour and halitosis before and after the switch were evaluated. Finally, a post hoc analysis was performed grouping hospitalizations according to the reason of admission (disease related, transplant related, possibly medication related and unrelated) and comparing them before and after the switch.

### Statistical analysis

A descriptive statistical analysis was performed for all the study endpoints. Continuous variables were summarized by several valid cases (*N*), mean, standard deviation (SD), median, interquartile range (IQR) and range values (minimum and maximum). Categorical variables were described by absolute and relative frequencies of each category over the *N*.

## RESULTS

### Study participants

Nine subjects diagnosed with NC were enrolled in the study, one female and eight males. The age at diagnosis ranged from 6 months to 6 years. The age of the patients at inclusion in the study was 21.95 ± 9.80 years (range 10.1–34.4), represented by three children, one adolescent and five adults (Table 1). The length of treatment with IR and ER cysteamine is described in Table 1. Due to age differences, disparities in disease progression between patients and a highly selected NC population, the clinical situation of those individuals was very heterogeneous. Therefore, two different populations were considered and evaluated separately based on age (five adult patients and four paediatric/adolescent patients).

**Table 1:** Patient population with NC and kidney characteristics at the study baseline

Patient	Sex	Age at inclusion (years)	Age at diagnosis (years)	eGFR (ml/min/1.73 m^2^)	KRT at switch	Age at first RRT (years)	Date of start of IR cysteamine	First visit reported (months)	Age at switch (years)	Last visit reported (months)	Reasons for switching	Extrarenal manifestations of NC	Concomitant pathologies at inclusion
1	M	12.1	0.7	112	No		1 June 2009	−36	8.1	36	Uncontrolled levels of WBC cystine, inability to sleep at night, bad body odour, halitosis, patient request	Neurocognitive alterations, corneal crystals, other	No pathologies
2	F	10.1	0.6	105	No		1 June 2009	−36	6.1	36	Uncontrolled WBC cystine levels, inability to get a good night's rest, difficulty in reconciling school or work, bad body odour, halitosis, patient request	FTT and impaired growth, photophobia, corneal crystals, other	No pathologies
3	F	11.1	1.3	82.17	No		27 January 2011	−36	7.0	36	Adverse effects (especially GI), other (lack of tolerance)	FTT and impaired growth, neurocognitive alterations, photophobia, corneal crystals, other	No pathologies
4	M	33.3	2.7	8	Kidney transplantation, dialysis	7.4, 22.7	1 October2012	−36	31.2	24	Lack of adherence to treatment	Hypothyroidism, diabetes mellitus, postnatal delay, photophobia, corneal crystals, retinal blindness, other	No pathologies
5	M	16.8	6.2	83	Kidney transplantation	8.2	NR	−36	13.9	36	Patient request	Corneal crystals	No pathologies
6	M	26.7	1.5	29	Kidney transplantation	20.4	1 March 1995	−36	24.7	24	Adverse effects [severe GI (diarrhoea) symptoms]	FTT and impaired growth, hypogonadism, fertility alterations, skeletal muscle motor function, other, photophobia, corneal crystals	Large B-cell non-Hodgkin's lymphoma (25 November 2015)
7	F	34.4	1.7	42.3	Kidney transplantation	10.0	5 November 2013	−36	32.4	24	Adverse effects (GI)	Hypothyroidism, hypogonadism, skeletal muscle motor function, orofacial motor function (speech and swallowing), musculo-respiratory function, other	Non-Hodgkin's lymphoma B diffuse large cell CD30^+^ stage III (1 January 2001), splenectomy (1 August 2001), right paramedial disc herniation D12–L1 (1 January 2016)
8	M	22.2	1.2	82.87	Kidney transplantation	14.7	1 December 1998	−36	19.2	36	Other (difficulty in reconciling NC treatment with immunosuppression), adverse effects of transplantation (immunosuppression of transplantation), adverse effects	FTT and impaired growth, central nervous system, other, photophobia, corneal crystals	Right nephrectomy for clear cell carcinoma (7 August 2018); cholelithiasis (10 October 2018), gastro-oesophageal reflux, migraines are pathologies concomitant to kidney disease or simply concomitant
9	F	31.0	0.7	109.3	Kidney transplantation	9.9	22 August 1990	−36	27.9	36	Lack of adherence to treatment	Hypothyroidism, growth delay, advanced myopathy (motor, oropharyngeal, swallowing, respiratory), neurocognitive	No pathologies

Reasons for change: in cases where several reasons are indicated, the one indicated first is the one considered as the main reason for the change by the researcher concerned.

NR: not reported; FTT: Failure To Thrive; NC: Nephropathic Cystinosis; KRT: Kidney Replacement Therapy; m: months; IR: immediate-release.

### Baseline characteristics

At baseline, three of four paediatric patients had a normal eGFR, whereas the adolescent received a KT at the age of 8 years (Table 1). All of them had no systemic manifestations of cystinosis except corneal crystal deposits and photophobia. Other common symptoms were failure to thrive (FTT), impaired growth and significant GI symptoms, in particular, patient 3 (Table 1), who required parenteral nutrition.

Regarding the adult participants (five patients), all of them had a KT and/or a dialysis history. Remarkably, three of the five KT patients had suffered from malignancies possibly related to prolonged immunosuppression and not to NC (Table 1). In addition, all adults presented extrarenal manifestations of cystinosis (Table 1), exhibited a baseline deteriorated clinical situation and had a history of poor IR cysteamine tolerance and adherence (patients 4, 7, 8 and 9; Table 1). In summary, this adult cohort represented a selected profile of NC patients with high disease severity and potentially poor past treatment compliance.

The main reasons for switching from IR cysteamine to ER cysteamine treatment are described in Table 1. Remarkably, more than one reason for switching treatment in single patients were often recalled. Concomitant medication received by patients is reported as Supplementary Fig. S1.

### Clinical results

**Figure 2:**
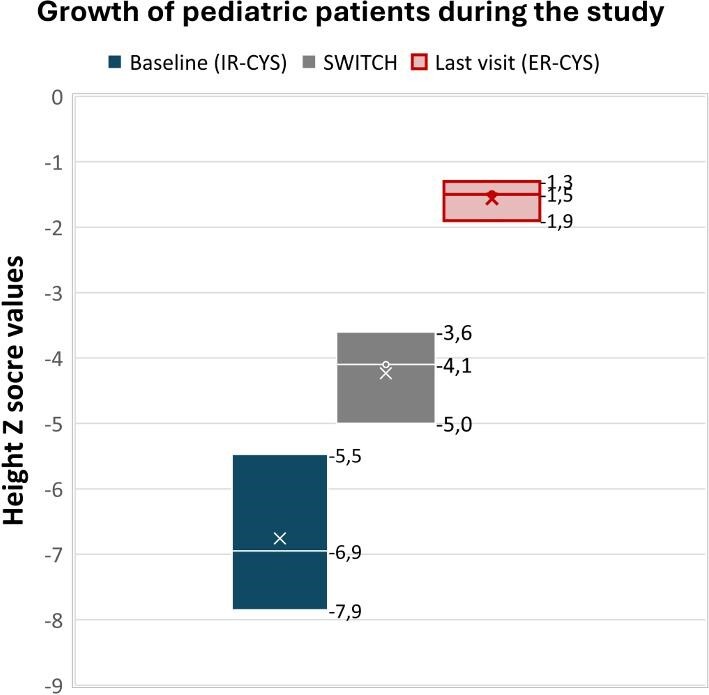
Growth in paediatric patients represented by height Z score at baseline (IR-CYS), at day of the switch and at the last visit (ER-CYS). The values in the blot plot correspond to percentile 25, median and percentile 75, from the bottom to the top.

After switching from IR cysteamine to ER cysteamine, growth improved in all paediatric groups throughout the observational period (Fig. 2). Two children who received growth hormone remained on a stable dose after the switch. Further, we also observed weight gain in the entire paediatric group and in the youngest adult (patient 8) after the switch as an expression of better nutritional status (data not shown).

**Figure 3:**
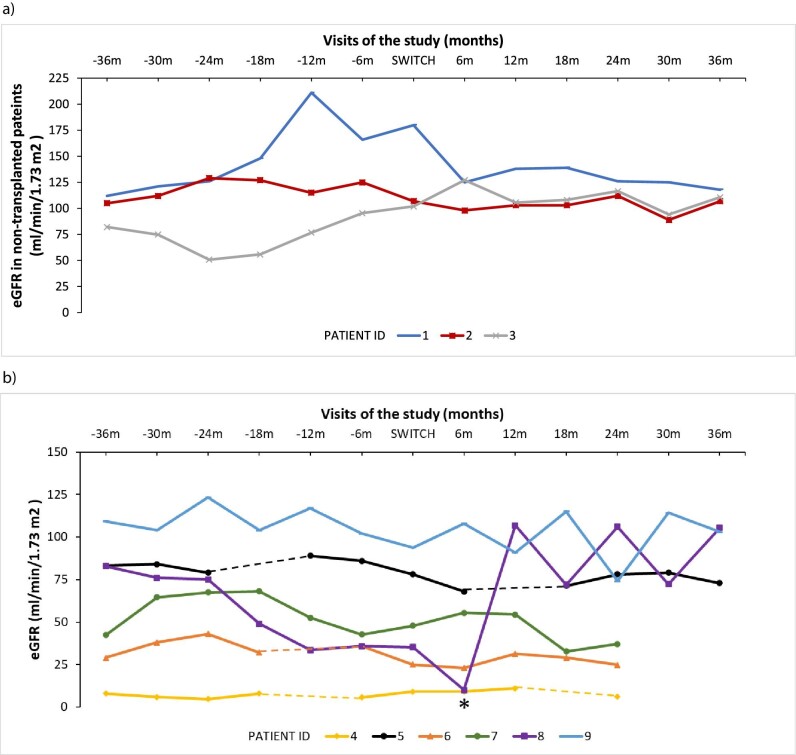
Kidney function based on eGFR during the study for (**a)** non-transplanted patients and (**b)** transplanted patients. In case of missing values, the dots are linked by discontinuous lines, which do not represent the real curve for those patients. *Patient transplanted at visit 6 months after the switch. Pt: patients; m: months. In paediatric patients, eGFR was measured using the modified Schwartz formula; in adult patients, eGFR was measured using the CKD-EPI formula.

Regarding kidney function, patients with a stable eGFR before the switch maintained or improved their eGFR after the switch to ER cysteamine in transplanted and non-transplanted patients (Fig. 3a and b).

**Figure 4:**
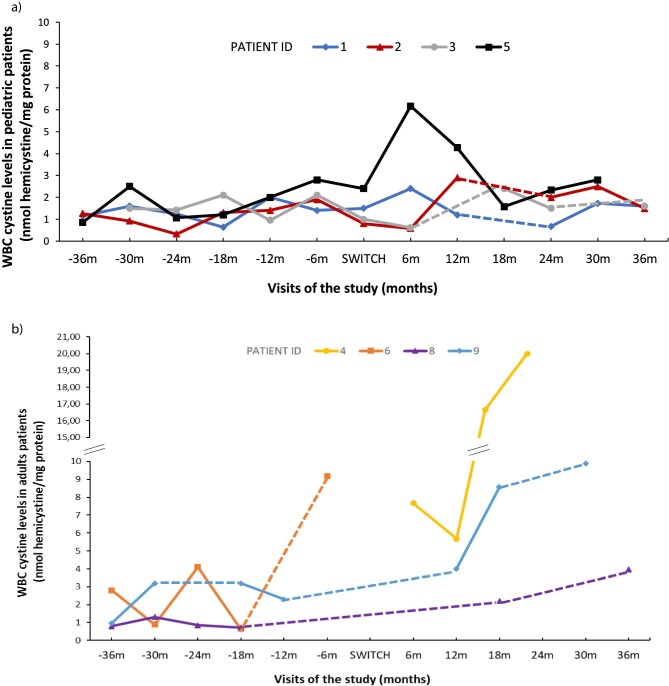
WBC cystine levels in (**a)** paediatric patients and (**b)** adult patients during the study (only values considered reliable by the investigator are represented). In case of missing cystine values, the dots are linked by discontinuous lines, which do not represent the real curve of WBC cystine levels for those patients. The cystine level of patient 7 was reported for only one visit. Pt: patients; m: months.

Overall, no significant differences in WBC cystine levels were observed before or after the switch. However, except for patient 5, the paediatric group (Table 1, Fig. 4a) maintained WBC cystine levels within the target therapeutic range throughout the observation period, independent of the type of cysteamine prescribed. In contrast, in the adult population, many patients did not have cystine levels reported or were considered unreliable by the treating physicians (Fig. 4b). Further, it was observed that some adults with elevated WBC cystine levels received subtherapeutic doses of cysteamine throughout the study, and independent of the type of cysteamine prescribed, patients receiving <50% of the recommended dose had elevated WBC cystine levels, as expected (Table 2, Fig. 4).

**Table 2:** Dose of cysteamine (g/m^2^/day) received by each patient at every study visit

Patient	−36 m	−30 m	−24 m	−18 m	−12 m	−6 m	Switch	6 m	12 m	18 m	24 m	30 m	36 m
1	1.82	2.08	1.73	1.73	1.95	1.95	0.87^a^	1.25^a^	1.52	1.63	1.63	1.44	1.44
2	1.63	1.56	1.56	1.82	1.82	1.82	0.98^a^	1.50	1.63	1.63	1.41	1.52	1.57
3	0.57^a,b^	1.04^a^	1.30	1.30	1.08^a^	1.56	0.78^a^	0.65^a^	0.65^a^	0.65^a^	0.70^a^	0.70^a^	0.93^a^
4	NA	0.10^a,b^	0.10^a,b^	0.10^a,b^	NA	NA	0.07^a,b^	0.36^a,b^	0.26^a^,^b^	0.39^a,b^	0.20^a^,^b^	NA	NA
5	1.39	1.39	1.46	NA	1.46	1.54	0.51^a,b^	0.87^a^	NA	1.19^a^	1.19^a^	1.19^a^	1.30
6	1.56	1.56	1.56	1.56	1.56	0.52^a,b^	1.46	1.66	NA	1.76	1.85	NA	NA
7	NA	NA	0.43^a,b^	0.43^a,b^	0.43^a,b^	0.43^a,b^	0.22^a,b^	0.22^a,b^	0.43^a,b^	0.43^a,b^	NA	NA	NA
9	0.33^a,b^	0.88^a^	0.88^a^	0.98^a^	0.88^a^	0.88^a^	NA	0.88^a^	0.88^a^	0.88^a^	0.93^a^	0.93^a^	NA
10	0.98^a^	0.98^a^	0.98^a^	1.10^a^	1.10^a^	0.98^a^	0.16^a,b^	NA	0.49^a,b^	0.61^a,b^	0.61^a,b^	0.61^a,b^	0.61^a,b^

The therapeutic range of cysteamine doses (as indicated in the summary of product characteristics): 1.3–1.95 g/m^2^/day; ^a^dose below the therapeutic range (<1.3 g/m^2^/day); ^b^dose below the therapeutic range (<1.3 g/m^2^/day) being also <50% of the minimum recommended dose.

NA: not available.

In terms of safety, this study looked at GI adverse effects, presence of body odour and/or halitosis and the use of PPIs. Five patients reported fewer GI effects while taking ER cysteamine, two patients described no significant changes before and after the switch and two patients described an increase in the occurrence of GI adverse events. Remarkably, patients who showed a marked weight improvement after the switch were those who also showed fewer GI adverse effects, possibly due to better treatment tolerability. Body odour was also reported less frequently in three of the four patients who had this complication before the switch, and complaints of halitosis were less frequent in all four patients who reported it before the switch. Finally, some of the patients reported less use of PPIs during the visits after switching treatments (Table 3, Supplementary Fig. S2a–d).

**Table 3:** Number of visits (*N*) with reports of GI effects, use of PPIs, halitosis and body odour

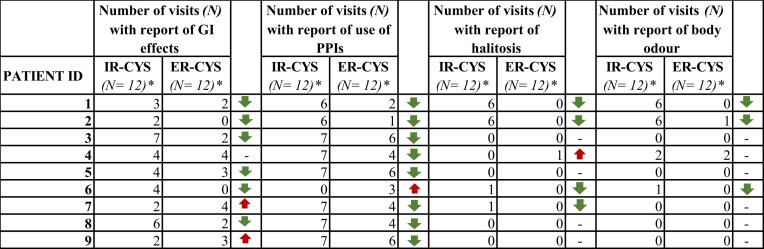

*A total of 12 visits (*N* = 12) corresponds to each period, from −36 months to 36 months.

**Figure 5:**
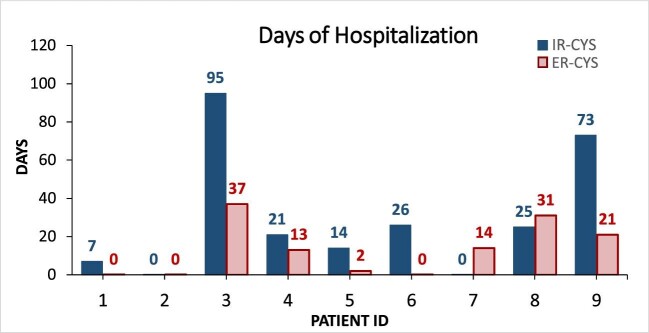
Duration (days) of hospitalization during the study period before and after cysteamine switch. Pt: patients; NR: no hospitalizations reported at that visit; VPRE: pre-switch visits; VPOST: post-switch visits.

One impressive study finding was a significant reduction in hospitalization duration observed in those patients admitted due medication or disease-related events (Fig. 5), with a decrease of 75% in hospitalization days after the switch to ER cysteamine. Thus admission length decreased from 29 ± 33.1 days to 13.1 ± 14.1 days on IR versus ER cysteamine, respectively, which represents a key positive outcome for patients and a potential savings in resources for the NHS (Fig. 5). Furthermore, we observed fewer hospitalizations (in terms of frequency) after switching to ER cysteamine, but differences were not significant.

## DISCUSSION

In a highly selected population of patients with NC, we demonstrated that switching from IR to ER cysteamine was associated with patient weight gain and, in paediatric patients, with growth improvement. Other study findings were a lower incidence of treatment-related adverse effects, such GI manifestations, and a reduction in patient admission episodes.

This study involved a cohort of nine heterogeneous but highly selected NC patients in Spain. Overall, at the time of the medication switch, enrolled patients had a history of significant adverse events, systemic disease involvement, comorbidities and frequent hospitalizations, mainly in adults. That is explained by the limited access to ER cysteamine in Spain, a medication not yet reimbursed but potentially available for compassionate use in severe cases. Thus ER cysteamine could be an opportunity to optimize primary cystinosis management in all clinical scenarios.

In this series, there were no statistical differences in WBC cystine levels comparing both oral cysteamine types. One plausible explanation is the fact that a subset of patients received <50% of the recommended daily cysteamine dose (described as the targeted maintenance dose and the maximum daily dose indicated in the Summary of Product Characteristics, 1.3 g/m^2^/day and 1.95 g/m^2^/day, respectively [20]), which may be attributed to GI intolerance. Three patients (4, 7, and 9; Table 2) taking a low cysteamine dose exhibited elevated WBC cystine levels, suggesting a possible correlation. In all three individuals, the suboptimal cysteamine dose was justified by patient adverse events, significant GI complaints and poor compliance. In addition, some adults had few WBC cystine level monitoring records or the results were considered unreliable by the investigator.

In contrast, in paediatric patients, the study findings showed a very positive impact on statural growth during ER cysteamine treatment, in agreement with previous reports [13]. The extent of impact on height was even greater than reported in previous studies in children treated with IR cysteamine at a mean age of 46 months, showing that those children maintained growth but did not reach normal height measurements compared with their peers [21]. As in Ahlenstiel-Grunow *et al*. [22], we also found a decrease in GI side effects in our study cohort. Also, patient 3, who was on parenteral nutrition due severe medication intolerance, no longer required it after the switch to ER cysteamine. In summary, we suggest that the positive impact on patient weight and height observed after switching to ER cysteamine can be explained by enhanced nutrition and increased intake due better GI tolerance.

Additionally, as reported by Ahlenstiel-Grunow *et al*. [22], those patients with preserved kidney function at the study baseline remained stable or with a higher eGFR after the switch to ER cysteamine. Nevertheless, the positive impact on eGFR observed in this study can be partially attributed to better hydration and improved patient clinical condition rather than to the cysteamine type itself, as seminal ER cysteamine studies demonstrated stable eGFR in treated patients [11]. Furthermore, one patient received a KT during the study (6 months after the switch), so it did not meet the classification used for the eGFR data. Although it could be a possible limitation of our study, we decided to represent transplanted and non-transplanted eGFR data separately because of the sample heterogenicity and the special characteristics of the patients.

In terms of safety, our study confirms the results of Ahlenstiel-Grunow *et al.* [22] not only regarding a decrease in GI side effects, but also reduced halitosis and body odour observed in most patients after switching to ER cysteamine. Similar results were also reported by Besow *et al.* [23] and in a substudy conducted by Greenbaum *et al.* [24], where treatment with twice-daily ER cysteamine caused less halitosis than treatment with IR cysteamine. This finding is key, given that halitosis makes it difficult for patients to comply with treatment and greatly negatively affects their self-perception and social relationships, especially in adolescents and adults, where it has been demonstrated that adherence decreases compared with paediatric patients [16].

However, not all patients in this study recalled fewer side effects. Indeed, some of them described more adverse events after switching to ER cysteamine, a finding that we attribute to the fact that they were indeed taking ER cysteamine compared with the earlier period characterized by poor adherence to IR cysteamine, if any. For instance, patient 4 received up to three times the ER cysteamine dose compared with the previous IR cysteamine dose (Table 2), which could also explain the increase of side effects during the ER cysteamine period compared with the IR cysteamine period. In line with our findings, Gaillard *et al.* [15] found that the dosing of ER cysteamine versus IR cysteamine (2 doses/day versus 4 doses/day, respectively) could result in better treatment adherence and improved QoL.

Remarkably, our study showed a significant reduction in hospitalization days, especially those episodes of admission related to the disease and those possibly related to medication side effects. Although ER cysteamine treatment currently costs more than IR cysteamine treatment, an improvement in disease control, with fewer side effects and a reduction in hospitalizations, may likely result in significant cost savings, a finding that was also highlighted by Ahlenstiel-Grunow *et al.* [22], and also in a significant increase in patients’ QoL.

Currently, few studies compare the benefits of ER cysteamine versus IR cysteamine, and rarely in adult patients Therefore, this study was of great interest to collect data on the use of ER cysteamine in real clinical practice in nephrology departments in Spain.

In conclusion, we describe the experience of switching from IR to ER cysteamine in a highly selected group of patients with NC in Spain. The main reason for the switch was the presence of medication-related adverse events, mostly GI symptoms, and therefore reduced tolerability, as well as a lack of compliance and poor control of the disease. Our findings support that switching from IR cysteamine to ER cysteamine in clinical practice could help to improve tolerability and reduce hospital stays in patients with NC, as well as achieving optimal nutritional status and growth in paediatric patients. Furthermore, the dosing of ER cysteamine could promote therapeutic compliance and positively impact patient QoL.

### Study limitations

It should be noted that the limited number of patients minimizes the statistically significant results. In fact, NC is a rare disease, and it is challenging to collect data from a large sample of patients. Additionally, access to ER cysteamine in Spain is limited, which explains the small, treated sample available for clinical research.

As a retrospective study, data collection is limited and may be unreported. Thus this study may not reflect the totality of patient experience and disease history. Finally, based on expert opinion, our study patients represent a severely impacted cohort within the NC population, which does not reflect the reality of patients with NC.

## Supplementary Material

sfae049_Supplemental_Files

## Data Availability

At this time, we will approve or deny data requests from external parties on a case-by-case basis. Chiesi reserves the right to deny requests for any and all legally appropriate reasons. Data requests that risk sharing participant-level data or proprietary information will not be approved.
