# Supplementary material for: Switching from immediate- to extended-release cysteamine in patients with nephropathic cystinosis: from clinical trials to clinical practice
Source: Clin Kidney J. 2024 Mar 6;17(4):sfae049. doi: 10.1093/ckj/sfae049 (PMC11022652; doi:10.1093/ckj/sfae049)
Supplement: sfae049_Supplemental_Files [file sfae049_supplemental_files.zip › Supplemetary_material_Figure_S1_CKJ.docx]

**Supplementary material**

**Figure S1. Concomitant medications**

Angiotensin-converting enzyme inhibitors: ACEi; Angiotensin (ii) receptors antagonists: ARA

Others: vitamin and food supplements, diuretics, antiemetics, laxatives, antihypertensives), antiplatelet agents, oral antidiabetics, calcium antagonists, anti-infectives, antibiotics, anti-ulcer drugs, anxiolytics, antipsychotics, bronchodilators, proton pump inhibitors, lipid lowering agents, prokinetics, antiepileptics, contraceptives, antianaemics, corticosteroids, uricosurics, gallstone-dissolving agents, drugs for the treatment of alopecia, and oxygen.
